# Akinetopsia: a systematic review on visual motion blindness

**DOI:** 10.3389/fneur.2024.1510807

**Published:** 2025-02-10

**Authors:** Johanna L. Browne, Lydia Krabbendam, Jan Dirk Blom

**Affiliations:** ^1^Faculty of Behavioural and Movement Sciences, Free University, Amsterdam, Netherlands; ^2^Parnassia Psychiatric Institute, The Hague, Netherlands; ^3^Faculty of Social and Behavioural Sciences, Leiden University, Leiden, Netherlands; ^4^Department of Psychiatry, University of Groningen, Groningen, Netherlands

**Keywords:** agnosia, Alice in Wonderland syndrome, metamorphopsia, visual distortion, visual motion perception

## Abstract

**Background:**

Akinetopsia, or visual motion blindness, is a perceptual distortion characteristic of Alice in Wonderland syndrome in which people see moving objects as disjointed ‘jumps’ or ‘freeze frames.’ Despite its profound impact, the condition remains poorly understood beyond the established centrality of cortical area V5/MT in visual motion perception.

**Methods:**

We carried out a systematic review of case descriptions on motion blindness, including those where additional symptoms were described.

**Results:**

Our search yielded data on 25 clinical and 27 experimental cases, the latter induced by cortical stimulation. Of the clinical cases, 12% showed hemiakinetopsia, 50% continuous or progressive symptoms, and 52% a chronic course. Pathophysiologically, in right-handed individuals, the left area V5/MT was found to be particularly susceptible to acute interference, as evidenced by the experimental studies. In contrast to earlier studies, we found a greater prevalence of right-hemispheric afflictions in clinical cases, suggesting that the right area V5/MT plays a more dominant role in motion perception. Bilateral lesions to V5/MT most often coincided with global akinetopsia and chronicity, although we found that the severity of the condition also depends on surviving components of the visual motion network as a whole, and—in line with the dynamic parallellism theory—the speed of moving objects. Aetiologically, most cases were associated with structural neurological conditions such as stroke and neurodegenerative disease, and fewer with intoxications or paroxysmal neurological disorders such as epilepsy. Treatments were mostly successful when they were aimed at the underlying condition, while cases due to organic lesions tended to be therapy-resistant. Although individual reports confirm that akinetopsia may have detrimental effects on people’s lives, data were insufficient for a proper analysis of such experiential aspects.

**Conclusion:**

Phenomenologically, pathophysiologically, and etiologically, akinetopsia is more heterogeneous than previously thought. We provide recommendations for clinical practice and further scientific research.

## 1 Introduction

‘Life is motion’ is the title of an early poem by the 20th-century poet and philosopher, Wallace Stevens (1). If there is any truth to that, what would it mean to be suffering from motion *blindness*? Wouldn’t that be a crisis of truly existential proportions? This rare condition, also known as visual motion blindness or akinetopsia, involves a selective deficit in the ability to visually perceive movement (2). Taken for granted by most of us in everyday life, visual motion perception crucially enables us to detect and analyse changes in optical information across spatial and temporal dimensions, thus allowing us to interpret and make sense of dynamic cues, and helping us to judge the speed and direction of stimulus movement (3–5).

Akinetopsia may manifest suddenly or gradually, either as an isolated condition or as part of a broader complex of phenomena (6, 7). It involves an absent or strongly diminished capacity to discern the smooth movement of objects. Instead, people tend to see moving objects as freeze frames or still-shots that make jumps between locations, akin to a stroboscope’s effect (8, 9). They report that birds in flight appear disjointed, people walking down a flight of stairs materialise in ever lower positions, and liquids appear frozen mid-pour like a glacier (10–12). Since visual motion perception serves a critical role beyond mere perception, deficits influence our daily lives and shape our experiences and interactions in profound ways. Routine activities like walking, using public transport, watching television, reading, socialising, and cooking become challenging and sometimes nigh impossible (10, 12–15). What is more, our safety and chances of survival hinge on this skill in uncountable instances involving depth perception, transportation, sports, functional activities such as catching and reaching, and even nonverbal communication (e.g., interpreting gestures and body language; lipreading) (5, 8, 12, 13, 15–17). These examples underscore the centrality of visual motion perception in ensuring our everyday safety and well-being. And yet, despite this centrality, impairments are not always recognised for what they are by health professionals or even by the people experiencing them. The main reason for this is that the condition is not widely known, even among neurologists, psychiatrists, and ophthalmologists, the medical specialists most likely to encounter it in clinical practice. This is complicated further by the fact that people with akinetopsia may have a hard time verbalising what is going on, and may therefore never show up in the consulting rooms of said specialists (10).

Perhaps as a consequence, the number of publications on akinetopsia is still scarce. A recent review counted 16 unique cases, published on over the past 40 years, meaning that on average, only a singe case had been published every 2.5 years (18). Arguably, most insights stem from the extensive research carried out in a single person experiencing the condition, addressed in the literature as ‘L.M.’ Zihl et al. (12) initiated this exploration, and in 1991 Zeki (7) was the one who coined the term akinetopsia. During the 1980s, the seminal publications on L.M. led to a swift acceptance of akinetopsia as a distinct neurological phenomenon in the scientific community, and profoundly altered our ideas on the neurological mechanisms underlying visual motion perception (15, 19). With hindsight though, L.M.’s case was certainly not the first to have been published on this intriguing phenomenon. Akinetopsia *avant la lettre* had previously been described in war-time publications such as those by Wagner (20), Hoff and Pötzl (21), and Goldstein and Gelb (22), with the first account possibly dating back to 1911 (23, 24). It may even be that the famous German magistrate Daniel Paul Schreber (1842–1911) sought to describe akinetopsia in his autobiographical *Memoirs of My Nervous Illness* of 1903, where he frequently referred to people seemingly popping up at random, designated by him as *flüchtig hineingemachte Männer* [‘fleetingly improvised men’ (25)]. These early publications were hardly noted though, and it was the flurry of publications on L.M. instead that moved akinetopsia into the centre of scientific attention. The name given it by Zeki (7) moreover helped to secure its status as a nosological concept.

### 1.1 Demarcating the field

Akinetopsia can be experienced in the absence of any other visual impairments such as those of visual acuity, contrast sensitivity, and the preception of colour (19, 26). In terms of descriptive pathology, it is considered a visual distortion rather than a hallucination (where something is perceived that is not there) or an illusion (where an actual object is perceived as something else, such as a face seen in the pattern of a rug or a tree trunk) (2). As a consequence, it is classified as one of the >40 visual distortions (or metamorphopsias) that fall under the umbrella term of Alice in Wonderland syndrome (27). Phenomenologically and pathophysiologically, akinetopsia should be differentiated from related perceptual distortions such as tachypsychia (the speeding up or slowing down of perceived time in accordance with one’s overall mental and physical state), the Zeitraffer phenomenon (a pathological sense of time speeding up), the Zeitlupen phenomenon (a pathological sense of time slowing down), and motion-induced blindness (23). The Zeitraffer and Zeitlupen phenomena are classified as time distortions, and likewise considered characteristic of Alice in Wonderland syndrome. Motion-induced blindness, unlike visual motion blindness, is an attentional phenomenon where salient objects intermittently shift into and out of awareness when overlaid on global moving patterns (23, 28). Distinguishing these phenomena from another and akinetopsia is crucial as their different underlying mechanisms hold important implications for both diagnosis and treatment.

### 1.2 Etiopathology

The fact that akinetopsia can be experienced in isolation is considered proof that the brain treats motion as a distinct aspect of vision (11). Although the mechanism underlying it is not fully understood, a crucial role in the encoding of visually perceived movement is attributed to the middle temporal area (MT or area V5) and the adjacent medial superior temporal area or MST (collectively known as MT+) (29). These areas specialise in the assessment of speed and direction, and are located bilaterally near the lateral temporo-parieto-occipital junction (30, 31). It has been suggested that akinetopsia arises primarily due to bilateral damage to—or dysfunctioning of—area V5 (16, 32–36). Such deficits can arise from a variety of structural lesions, typically ischemic or traumatic in nature, although paroxysmal neurological conditions such as epilepsy have also been reported (11, 37–40). While bilateral V5 damage is often considered a necessary condition for akinetopsia to arise, case reports suggest that unilateral hemispheric afflictions may suffice. However, some authors consider such cases subtler in nature, or construe them as being limited to the contralateral visual field (‘hemiakinetopsia’), leaving motion perception in the ipsilateral hemifield intact (6, 14, 32, 34, 35, 37, 39, 41–43).

**Figure 1:**
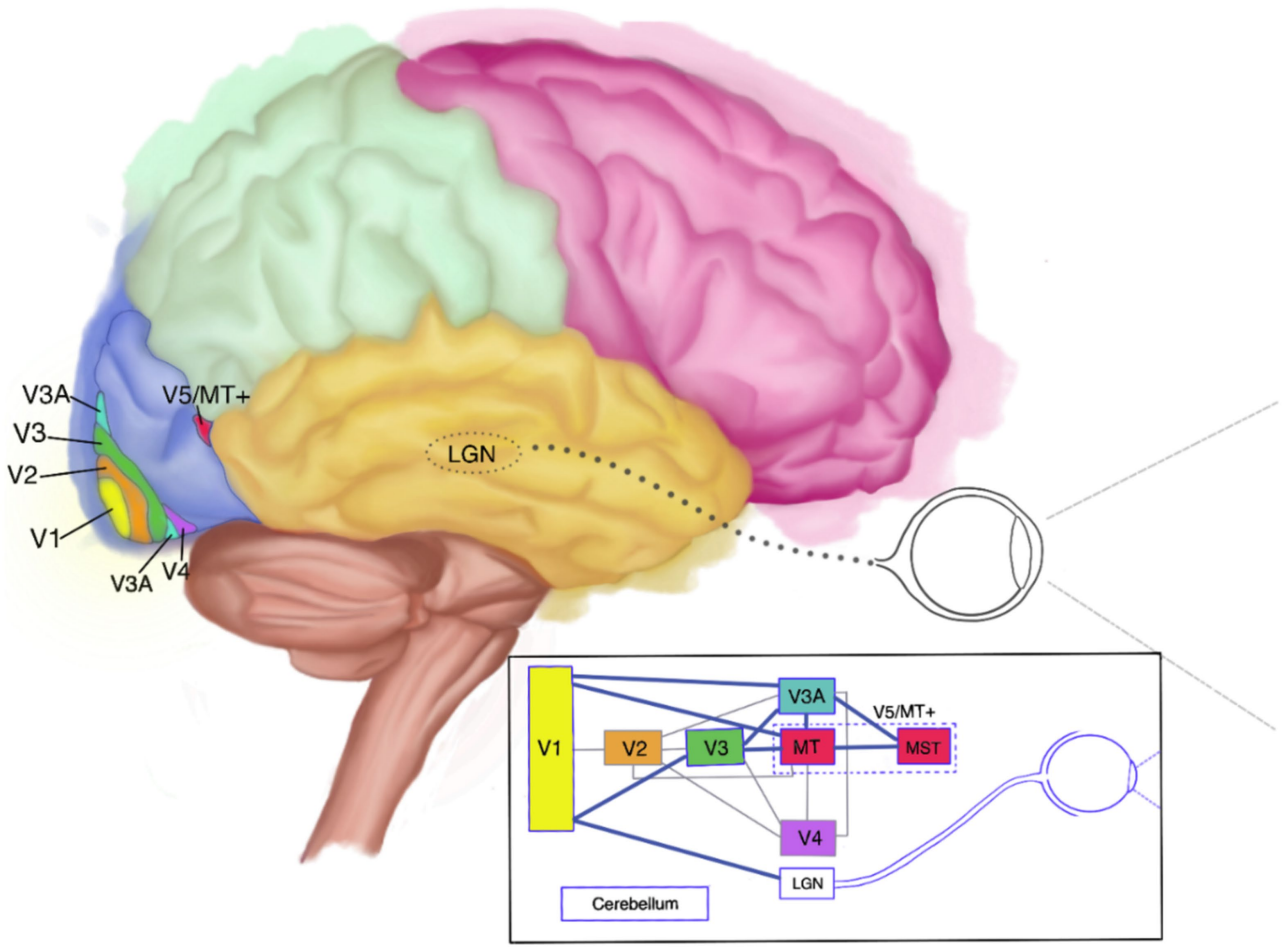
Anatomical and schematic depiction of the visual motion-perception network (side view). LGN, lateral geniculate nucleus.

In all, there is a fairly high degree of agreement on the primary cortical areas implicated in (impaired) visual motion perception, although the exact nature of the broader network involved is still shrouded in veils (44, 45). As far as currently known, this network encompasses interconnected cortical areas such as the lateral geniculate nucleus (LGN), V1, V3, the cerebellum, and MT+ (29, 46–50). It is generally believed that surviving components of that network may compensate for some motion perception deficits (4, 32, 47, 51). Since approximately 10% of the neurons located in V1/V2 have been found to be direction-selective, it is believed that especially those may contribute to residual motion perception in cases of V5 impairment. This may well have been the case with L.M., who showed a preserved ability to perceive slow-moving objects (4, 47, 51). All this indicates that the degree and severity of akinetopsia is not only linked to the type of affliction, but, importantly, also to parts of the wider network that may still be functioning. It moreover indicates that people may experience akinetopsia for certain velocities and not for others, and for the visual field as a whole or only part thereof (4, 15, 42, 47, 52). An anatomic and schematic depiction of the cortical areas involved in visual motion perception and their interconnections can be seen in Figure 1. In the schematic diagram included in Figure 1, the visual motion-perception pathway is shown by the thick blue lines. The thinner grey lines indicate other pathways involved in visual perception, but not believed to be directly involved in motion perception.

### 1.3 Clinical aspects

Given the scarcity of documentation on akinetopsia, standardised protocols for diagnosis and treatment have not yet been developed. Primarily, practice-based treatments as described in the literature tend to address the underlying aetiological factor rather that the impaired motion perception itself. The efficacy of other interventions for akinetopsia, such as visual motion perception rehabilitation or developing compensatory strategies, are only infrequently reported on. As far as we know, even the burden of akinetopsia has gone largely unexplored, with practical support for this debilitating condition being developed only in a haphazard way.

### 1.4 Aim

In the current systematic review we aim to summarise what is known about akinetopsia and address the knowledge gaps outlined above. With it, we intend to raise awareness and stimulate interest in this field to encourage further scientific research with the ultimate goal of facilitating and improving diagnosis, treatment, and support for this small and underserved group (which in reality may well be larger than we currently know).

## 2 Methods

### 2.1 Literature search

For the purpose of the present review we carried out a systematic literature search in PubMed, Embase, PsycINFO, Google Scholar, and the historical literature up until October 1, 2024. Filters were set to anytime and all types of papers; language was restricted to English, Dutch, and German. The primary keywords used were ‘akinetopsia’ and ‘motion blindness’. Based on the articles found, related search words were identified from the thesaurus issued by the National Library of Medicine and were added to the MeSH terms, e.g., ‘visual agnosia’, ‘visuospatial agnosia’, and ‘motion-induced blindness’. Despite having distinguished the latter phenomenon from motion blindness in our introduction, we thought it relevant to include the term in our search in an effort to overcome the variability in terminologies used to describe akinetopsia, and to thus include as many relevant cases as possible. We implemented these terms and their variations in our search, and supplemented the digital searches with reverse searches by examining the references of the relevant literature. Papers were only considered eligible for inclusion when they contained case descriptions or case series on akinetopsia, including experimentally induced cases [e.g., with the aid of transcranial magnetic stimulation (TMS)]. Importantly, we included all studies that reported on akinetopsia, even when other symptoms co-occurred. We thus aimed to maximise the number of empirically available data and thereby provide a more nuanced representation of the condition’s complexity and heterogeneous nature. Publications were excluded when they did not align with our eligibility criteria or described related, but different phenomena (e.g., time distortions, motion-induced blindness).

### 2.2 Data extraction

The data extracted from each text comprised, as far as available, (i) demographics (i.e., gender, age at symptom onset, handedness), (ii) phenomenological characteristics of akinetopsia (e.g., frequency of symptoms, duration), (iii) aetiology, (iv) hemispheric lateralisation, (v) impacted visual field, (vi) impact on well-being, (vii) diagnostic procedures used, (viii) treatments and other interventions applied, and (ix) outcome. Of note, we operationalised symptom frequency as either *continuous* in nature (persisting without interruption), *progressing* (with symptoms worsening over time), *transient* (with symptoms having a sudden onset and rapid resolution in the order of hours or days), *sporadic* (with symptoms occurring at irregular intervals) or *improving* (with symptoms showing improvement over time, usually gradually). Since symptom duration varied widely, consequently individuals were consolidated into the categories of *1–10 days*, *11–30 days*, *31–150 days*, *>150 days*, or *progressive* (for cases with worsening and indefinite symptoms). From the experimental studies we also extracted (x) stimulus methods and parameters. Given the small sample we mainly used descriptive statistics, and occasionally MATLAB (53).

## 3 Results

### 3.1 Search results

**Figure 2:**
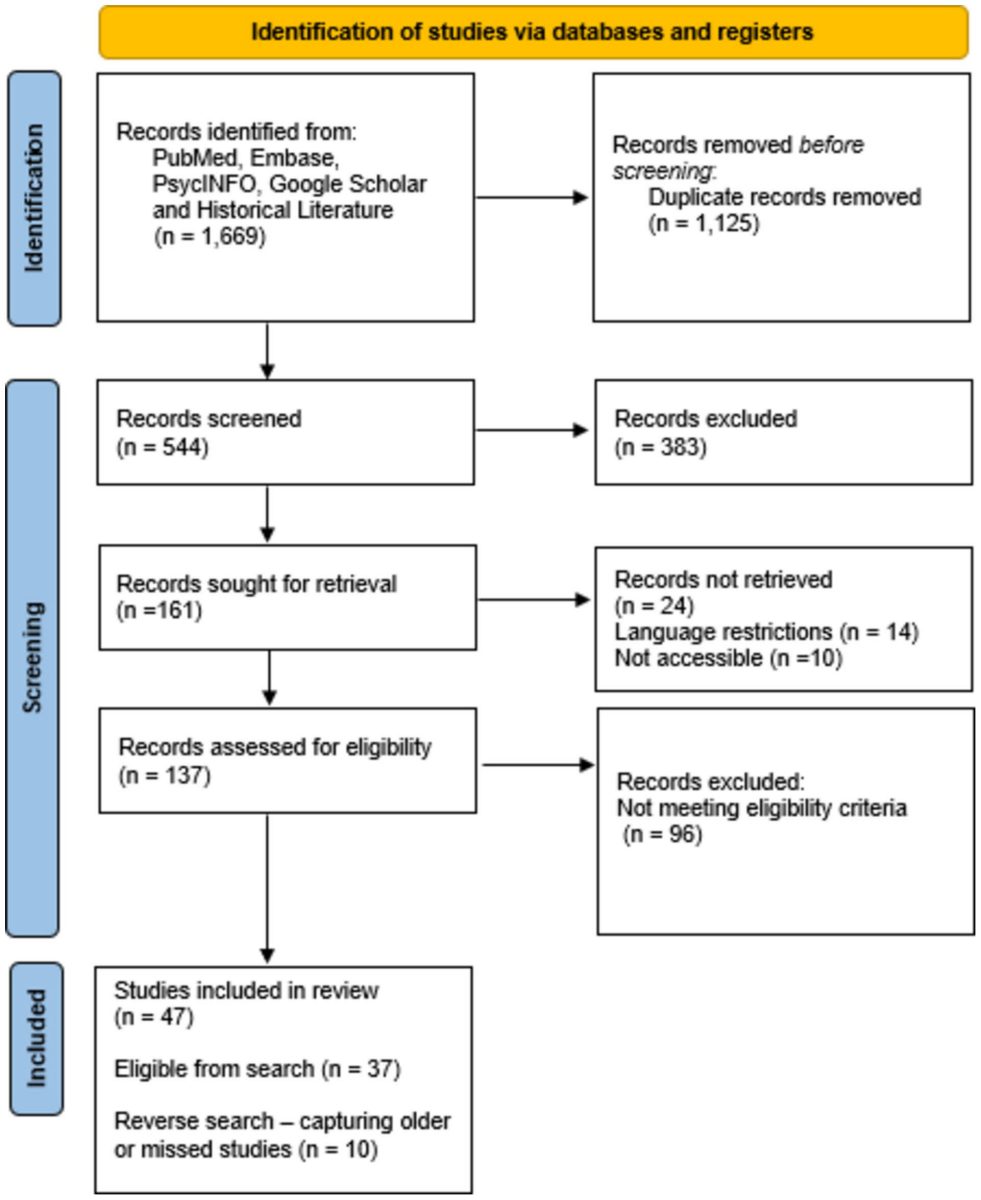
PRISMA flow diagram.

The search terms defined by us yielded 1,669 hits across all databases. After removing 1,125 duplicate records and excluding 383 publications for not meeting the eligibility criteria when screening titles and abstracts, the remaining 161 texts were sought for retrieval. Of these, 24 reports could not be retrieved due to language restrictions (*n* = 14) or inaccessibility (*n* = 10). The remaining 137 reports were assessed for eligibility, after which 96 additional records were excluded as they did not meet our eligibility criteria. As a result, 47 studies were included in the final review. This total comprised 37 studies identified from the database search and 10 studies added through reverse searches, which helped capture older or missed studies (Figure 2 depicts the PRISMA flow diagram).

In this collection of 47 clinical texts, 25 unique individuals with akinetopsia had been described, with L.M. being the most frequently reported case (for case details, see Supplementary Table S1 as well as Supplementary Table S2). Additionally, we identified 27 reports of experimentally induced motion blindness, caused either by invasive electrical stimulation (e.g., during awake brain surgery, *n* = 2) or TMS (*n* = 25) (see Supplementary Table S3 for a detailed overview). By applying inclusion criteria that did not rule out cases with co-occurring symptoms, we captured a broad spectrum of cases and studies. Thus we included a total number of 52 individuals with either clinical or experimentally-induced akinetopsia in our analysis. It is noteworthy that some case descriptions were reconstructed from multiple publications on the same individual.

### 3.2 Attributes of akinetopsia

#### 3.2.1 Clinical cases

The demographic and aetiological characteristics of the 25 clinical cases that we analysed are summarised in Table 1; for a more comprehensive overview, see Supplementary Tables S1, S2. The age of onset of akinetopsia varied from 19 to 73 years, with a mean age of 50 years. Of the 25 people analysed, 44% were female. Handedness data were available for a subset of eight persons (32%), of whom 87.5% were right-handed (*n* = 7), 12.5% left-handed (*n* = 1), and none ambidexter. As to phenomenology, 44% (*n* = 11) of the people reported on exhibited akinetopsia that affected the entire visual field, and 12% (*n* = 3) hemiakinetopsia. Underlying aetiologies had been established in 92% (*n* = 23) of the cases reviewed. They included stroke (28%, *n* = 7), epilepsy (12%, *n* = 3), neurodegenerative disease (specifically posterior cortical atrophy; 16%, *n* = 4), nefazodone toxicity (8%, *n* = 2), and traumatic brain injury (12%, *n* = 3). Four cases (16%) were due to other causes, comprising surgery, subcortical hemorrhage, Creutzfeld-Jakob disease, and brain metastases (each *n* = 1). As to pathophysiology, 84% (*n* = 21) of the reports had specified an affliction as involving some combination of the parietal, occipital, and temporal lobes. Five case reports (20%) detailed the involvement of area V5/MT. An analysis of lesion lateralisation showed that 48% (*n* = 12) of the people described had had bilateral lesions. Of the nine cases with unilateral lesions, 14% (*n* = 3) involved the left hemisphere, 29% (*n* = 6) the right hemisphere.

**Table 1:** Clinical cases of akinetopsia (*n* = 25): demographics and etiopathology.

Descriptives	All cases		Bilateral hemispheric affliction		Right unilateral		Left unilateral		N/A	
*N* = % of Total	25	100%	12	48%	6	24%	3	12%	4	16%
Age (onset)										
Mean	~50		53		~48		49		~45	
Range	19–73		20–73		19–60		24–68		~20–61	
Gender										
Male	14	56%	4		3		3		3	
Female	11	44%	5		3				1	
Handedness										
Right	7	28%	4		3					
Left	1	4%	1							
Ambidexter										
Not specified (N/A)	17	68%	7		3		3		4	
Aetiology										
Stroke	7	28%	4		2		1			
Epilipsey/Seizure	3	12%	1		2					
Neurodegeneration (PCA)	4	16%	3						1	
Substances	2	8%							2	
Trauma	3	12%	2				1			
Other	4	16%	1		2		1			
N/A	2	8%	1						1	
Lobules Affected										
Occipital	3	12%	3							
Temporal	1	4%			1					
Parietal	2	8%	1						1	
Occipito-Temporal	4	16%	2		2					
Temporo-Parietal	2	8%			2					
Parieto-Occipital	6	24%	3		1		2			
Temporo-Parieto-Occipital	3	12%	3							
N/A	4	16%					1		3	
V5/MT Affected										
Yes	5	20%	2		2		1			
No	1	4%					1			
N/A	19	76%	10		3		2		4	
Visual hemifield affected										
Bilateral	11	44%	6		4		1			
Left	2	8%	1						1	
Right	1	4%					1			
N/A	11	44%	5		2		1		3	
Symptom duration										
1–10 days	1	4%			1					
11–30 days	2	8%			1				1	
31–150 days	1	4%	1							
≥150 days	11	44%	6		3		1		1	
Progressive	4	12%	3						1	
N/A	6	24%	2		1		2		1	
Symptom frequency										
Transient	3	12%			1				2	
Sporadic	4	16%	1		2		1			
Improving	1	4%			1					
Continuous	10	40%	7		1		1		1	
Progressing	4	16%	3						1	
N/A	3	12%	1		1		1			

Table 2 provides details on the frequency and duration of symptoms, as well as on the visual hemifields affected. Half of the patients had reported on having continuous (40%, *n* = 7) or progressing symptoms (16%, *n* = 4), while 12% (*n* = 3) had reported transient symptoms, 16% (*n* = 4) sporadic symptoms, and 4% (*n* = 1) gradual improvement. Three case reports (12%) did not specify symptom frequency. The duration of akinetopsia ranged widely, from 5 days to over 30 years. The majority of cases reviewed (44%, *n* = 11) mentioned symptoms persisting >150 days. All individuals in this category had experienced symptoms for more than 5 months, with some cases describing symptoms exceeding 1 or 2 years, 10 years, and even over 30 years. One person (4%) had experienced symptoms for 31–150 days, two people (8%) for <30 days, and one (4%) for <10 days. In 12% of the cases symptom duration was not mentioned. The impacted visual fields were specified in 56% of the cases (*n* = 14), with 44% (*n* = 11) experiencing akinetopsia in both hemifields; of these, 55% (*n* = 6) showed bilateral cortical involvement, 9% (*n* = 1) left unilateral involvement, and 36% (*n* = 4) right unilateral involvement. Two people (8%) had described symptoms restricted to the left visual field (one of them accompanied by bilateral hemispheric lesions, the other one unspecified), and one other person (4%) had described symptoms restricted to the right visual field (in combination with a left hemispheric lesion).

**Table 2:** Clinical cases of akinetopsia (*n* = 25): symptom patterns and involvement of visual fields.

Descriptives	All cases		Bilateral hemispheric affliction		Right unilateral		Left unilateral		N/A	
*N*=% of Total	25	100%	12	48%	6	24%	3	12%	4	16%
Symptom frequency										
Transient	3	12%			1				2	
Sporadic	4	16%	1		2		1			
Improving	1	4%			1					
Continuous	10	40%	7		1		1		1	
Progressing	4	16%	3						1	
N/A	3	12%	1		1		1			
Symptom duration										
1–10 days	1	4%			1					
11–30 days	2	8%			1				1	
31–150 days	1	4%	1							
≥150 days	11	44%	6		3		1		1	
Progressive	4	12%	3						1	
N/A	6	24%	2		1		2		1	
Visual hemifield affected										
Bilateral	11	44%	6		4		1			
Left	2	8%	1						1	
Right	1	4%					1			
N/A	11	44%	5		2		1		3	

#### 3.2.2 Experimental cases

Six publications detailed studies on experimentally induced motion blindness, comprising a total of 27 participants. Details regarding age, sex, and handedness were generally lacking. All participants experienced brief and transient akinetopsia upon stimulation of area V5/MT. Two studies involved the application of cortical electrical stimulation (ES) on two 43-year-old female participants who were undergoing cranial surgery. The latter studies, conducted by Becker et al. (41) and Blanke et al. (54), will be denoted as study A and study B, respectively. The ES studies targeted V5, with one of them focusing on the left hemisphere (A), and the other on the right (B). For evaluation, the studies primarily used motion-direction discrimination tasks and verbal forced-choice paradigms. Results indicated that unilateral stimulation of area V5/MT induced motion blindness, impacting both contralateral and ipsilateral visual fields in case A. Additionally, study B noted that the direction-discrimination abilities of their participant varied depending on the movement direction of stimuli. The authors found that discrimination was weakened more in the ipsilateral (right) direction (63%) then in the contralateral (left) direction (19%), downwards (13%), and upwards (5%).

The remaining four studies used TMS to induce motion blindness in *n* = 25 participants. These studies, conducted by Beckers and Hömberg (55), Beckers and Zeki (56), Schenk et al. (57), and Walsh et al. (58), will be referred to as studies C, D, E, and F, respectively. These studies predominantly employed left unilateral hemispheric stimulation, apart from study C, which also utilised unilateral stimulation of the right hemisphere. Tasks performed during the experiments varied, but they included motion-direction discrimination, catching tasks, reach-to-grasp tasks, and search-array tasks. Most studies employed control or comparative sites, such as V1 (C & D) or neighbouring extrastriate areas (D & E). Stimulation of V5/MT consistently disrupted visual motion perception, with the left area V5 being particularly sensitive to this type of stimulation. Stimulation timing played a significant role, with optimal disruption occurring at specific intervals after visual stimulus onset (−20 to +10 ms). In one study, stimulation of V1 was also found to induce some visual motion perception deficits, at delays of 60–70 ms after stimulus onset. However, this was not nearly as potent as V5 stimulation. Further details and descriptives of these experimental studies can be found in Supplementary Table S3.

### 3.3 Mediating factors

#### 3.3.1 Hemispheric involvement

To assess the potential mediating role of afflictions to either the left or right hemisphere in the frequency, duration, and visual-field involvement of akinetopsia, we first analysed the data from the 25 clinical cases. Each variable was subdivided into categories. Hemispheric lateralisation was coded as either *non-applicable (N/A)*, *unilateral left*, *unilateral right, or bilateral*; frequency as *N/A*, *transient*, *sporadic*, *improving*, *continuous*, or *progressing*; duration as *N/A*, *1–10 days*, *11–30 days*, *31–150 days*, *>150 days*, or *progressive*; and visual-field involvement as *N/A*, *left*, *right*, or *bilateral*. Using these data, we constructed contingency tables or ‘heatmaps’ in MATLAB to illustrate the frequency distributions (Supplementary Figures S1–S3). In examining the interactions between lateralisation and frequency among people with continuous or progressive symptoms (*n* = 14), the majority (71.4%) exhibited bilateral hemispheric afflictions, while a smaller proportion presented with left unilateral or right unilateral afflictions (both 7.1%). For those with transient, sporadic, or improving symptoms, right unilateral hemispheric afflictions were predominant (50%), compared to bilateral and left unilateral afflictions (both 12.5%). Three case reports did not report on the frequency of symptoms (N/A). The distribution of symptom duration also varied, with 60% of the patients experiencing long-term symptoms (>150 days or ‘progressive’), primarily associated with bilateral afflictions (60%), and in 20% with right unilateral afflictions. Six of the 25 cases did not specify duration. As to the involvement of the visual fields, this was specified in 56% of the cases. Bilateral visual field impairment was reported in 44% of the cases, with bilateral hemispheric afflictions being the predominant correlate (54.5%), followed by right unilateral afflictions (36.6%).

Unlike these clinical cases, where affliction lateralisation and symptomology varied, the experimental cases were invariably characterised by transient and very brief episodes of akinetopsia, mainly brought about by unilateral cortical stimulation. Given the absence of diversity in symptom frequency and duration, for the experimental group we only carried out an analysis of hemispheric involvement in relation to visual-field impairment. None of the six studies employed bilateral hemispheric stimulation of V5. In four studies, V5 stimulation was conducted exclusively to the left hemisphere (41, 56–58). In stimulating V5 in the left hemisphere, Becker et al. (41) and Schenk et al. (57) reported that they induced akinetopsia bilaterally across hemifields, while Beckers and Zeki (56) found that left cortical TMS stimulation of V5 affected only the right (contralateral) visual field. Walsh et al. (58) did not specify the visual field affected by TMS stimulation despite reporting on successful motion-perception impairment in their participants. Beckers and Hömberg (55) applied TMS unilaterally to V5 in both hemispheres, with akinetopsia manifesting in the hemifield contralateral to the stimulation site. Blanke et al. (54) exclusively stimulated V5 in the right hemisphere, finding that they could induce visual motion perception deficits in the contralateral (left) visual field. In summary, 33.3% of the experimental studies found that left unilateral stimulation of area V5 induced bilateral visual-field impairments, and 50% affected only the contralateral visual field; 16.7% of the studies did not specify this.

#### 3.3.2 Velocity

Since akinetopsia may be observed for objects with certain velocities only, i.e., above or below certain speed thresholds, we had intended to conduct a correlation analysis on this topic. However, we were unable to do so because of limited data on this aspect in the clinical papers and the different ways in which velocity had been assessed across studies. We nonetheless gathered relevant data to explore this issue a bit further. Among the 25 clinical cases, five (20%) reported on object velocity, and showed that higher velocities tend to induce more severe motion-perception deficits. For example, L.M. perceived short-range motions below 6°/s correctly, struggled with velocities above 8°/s, and experienced akinetopsia at 12°/s. Two people referred to as A.F. and T.D. likewise experienced deficits with increasing velocities. Another person called M.B. reported impaired motion perception when looking at objects moving with a speed of 2°/s, while S.F. reported it at a speed interval of 11–17°/s. Across these cases, the mean threshold velocity for akinetopsia to be experienced was 11.9°/s (range: 2–16.5°/s). In all the experimental studies analysed by us, such object velocities had been established with the aid of motion-perception tests. The studies by Becker et al. (41), Blanke et al. (54), Beckers and Hömberg (55), Beckers and Zeki (56), and Walsh et al. (58) rendered object velocity in degrees per second (°/s), whereas the study by Schenk et al. (57) did this in metres per second (m/s). Table 3 shows an overview of the velocities found, which had a mean value of 4.1^o^/s, i.e., substantially lower than the mean velocity found in the clinical studies (and together combining to a mean velocity of 7.5^o^/s).

**Table 3:** Velocity thresholds of objects for akinetopsia.

Studies documenting velocity in akinetopsia presentation	Akinetopsia-inducing factor	Velocity threshold for akinetopsia
L.M (12, 17, 50, 72)	Stroke	6–8 ⁰/s
A.F (63, 68)	Stroke	<16.5 ⁰/s
T.D (73)	Stroke	~15 ⁰/s
M.B (16)	Stroke	2 ⁰/s
S.F (26, 73)	Seizure	~11–17 ⁰/s
(41)	ES	6 ⁰/s
(54)	ES	2 ⁰/s
(55)	TMS	4–9 ⁰/s
(56)	TMS	5 ⁰/s
(57)	TMS	~0.25–0.5 m/s
(58)	TMS	1 ⁰/s

### 3.4 Clinical aspects

#### 3.4.1 Impact on well-being

The burden caused by akinetopsia was rarely assessed in the literature reviewed. As a consequence, we were unable to perform any analyses. Nonetheless we singled out a few examples to illustrate the extent to which akinetopsia may affect people’s lives. M.B., for instance, had never had a dog phobia, but developed one because he no longer saw them coming and felt frightened when they were suddenly at his side (16). L.M. started avoiding situations where more than two people would be present, since, as she said, ‘*people were suddenly here or there but I have not seen them moving*’ (12, 15). A patient with undiagnosed akinetopsia became inactive and apathetic, chiefly lying on the couch, wasting away decades during which he was treated to no avail with all kinds of pharmacological agents—a time during which he might have built a career, started a family, and perhaps even seen his children off to start a life of their own (10).

#### 3.4.2 Diagnostic procedures

In the absence of any standardised tools, the diagnostic work-up of akinetopsia described tended to include history-taking, general physical, ophthalmologic, neurological, and psychological/psychiatric examinations, blood work, and auxiliary investigations such as a brain CT or MRI, an EEG, and sometimes psychophysical tests. These tests included ocular tracking, motion-direction discrimination, contrast sensitivity for moving striped patterns, random dot patterns, motion-speed discrimination, and tasks involving motion cues (6, 8, 16, 41, 48, 50). These often require advanced technology and expert interpretation. As noted, diagnosis may be thwarted by patient delay, which is not uncommon due to a lack of awareness of akinetopsia among health professionals and those affected, as seen in the clinical case reports by Blanke et al. (16), Blom (10), Burns (9), and Maeda (48). This may well be especially relevant in cases of hemiakinetopsia (32, 59).

#### 3.4.3 Treatment and other interventions

As to treatment, again insufficient data was available for a proper analysis. When detailed, studies tended to focus on addressing the underlying cause where possible, as is customary in the clinical management of Alice in Wonderland syndrome (10). Successes were notably observed for medications for epilepsy- and substance-induced deficits (39, 60, 61). In addition, several compensatory strategies were described, including avoidance behaviour and active adaptation (15). For instance, L.M. found that avoiding watching moving stimuli had a positive effect on guiding finger and hand movements, as well as on writing (12, 62). While walking in crowded places, linking arms gave her reassurance (15). Additionally, she developed an active adaptation strategy that involved prolonging observation times while relying on auditory cues and spatial displacement information of moving objects to estimate their velocity (12, 17, 57). Finally, some studies indicated potential for rehabilitation through restorative training of surviving components of visual motion perception (9, 32, 41).

## 4 Discussion

Our analysis of 25 clinical and 27 experimental cases of akinetopsia indicates that this seemingly unequivocal motion-perception disorder shows heterogeneity at several levels of conceptualisation. Starting at the phenomenological level, akinetopsia can impact either the full field of vision or just one half, with hemiakinetopsia reported on in 12% of the clinical cases. The speed threshold for moving objects to appear in a disjointed fashion was 7.5^o^/s on average, with most clinical studies reporting higher velocities and all experimental studies lower ones (probably due to greater measuring precision in controlled environments and the possibility to correct for compensatory mechanisms). We also found that half of the clinical group experienced continuous or progressive symptoms, with 52% of the whole group experiencing akinetopsia for half a year or longer. Due to the limited follow-up of most studies, this is probably an underestimate. As to the pathophysiological level, we found that the bilateral area V5/MT was of key importance to the mediation of akinetopsia, although motion perception clearly depends on the proper functioning of the motion-perception network as a whole. Testament to this is the sparing of motion perception for relatively low velocities, the possibility to experimentally induce akinetopsia by targeting V1, and a unique case of akinetopsia with sparing of area V5/MT where lesions to the inferior parietal lobe and parietal-occipital junction were held responsible for the ensuing deficit (11). Our analysis of lesion lateralisation indicated that bilateral hemispheric damage is more likely to result in continuous or progressive symptoms affecting the entire visual field than unilateral damage. In contrast, unilateral lesions, particularly on the right side, were initially thought to produce ‘milder’ symptoms. However, our findings revealed these unilateral lesions can not only cause visual deficits in one hemifield (hemiakinetopsia) but also induce motion blindness across the entire visual field, for an extended amount of time. Furthermore, the experimental studies showed that akinetopsia is induced most effectively by targeting the left area V5/MT. Therefore it may be that—in right-handed persons—the left area V5/MT is the one most vulnerable to acute interference, whereas its right-sided homologue is of vital importance in the long run. This is in need of further study, but an argument in favour of this hypothesis is that right unilateral lesions in the clinical group appeared to trigger akinetopsia in the whole visual field more often than left unilateral lesions (16% versus 4%). In all, this would seem to indicate that the right-sided area V5/MT has a more dominant role in visual motion perception. At the aetiological level we saw that most cases of akinetopsia are associated with structural neurological conditions such as stroke, neurodegenerative disease (particularly posterior cortical atrophy), and traumatic brain injury, but that the whole range of underlying disorders also included intoxications and paroxysmal neurological disorders. Treatment, whenever possible and detailed, was usually aimed at these underlying conditions. At the experiential level, finally, we found that the impact on people’s daily lives was assessed insufficiently in most studies to allow for a proper analysis, although individual case reports indicated that these were profoundly life-altering. Importantly, akinetopsia is bound to affect one’s emotional well-being, social functioning, and adaptive-learning capacities, and may lead to fear learning, phobias, passivity, and social isolation.

### 4.1 Implications for the visual motion-perception network

Our findings suggest a primacy of the non-dominant hemisphere in visual motion perception. This is in opposition to the conclusions drawn by van Swol et al. (18) in their recent review, and aligns with studies that implicate the right ventral visual cortex and the integrity of the dorsal pathway, including area V5, as critical for uncompromised motion perception (32, 46, 48, 63). However, it is as yet too soon to decide how firmly this conclusion can be drawn. After all, the experimental studies cited above revealed that left-hemispheric stimulation of V5 was followed by a greater reduction in performance than right-hemispheric stimulation. This finding is supported by earlier PET scan results (7), and highlights the need for further research to clarify the role of these two homologous areas. The clinical and experimental findings on object velocity here presented align with Beckers and Zeki (56), and with ffytche et al. (64), who demonstrated that varying speeds alter input to area V5, which is activated before V1 by fast-moving stimuli, and vice versa at smaller velocities. This is in line with the dynamic parallelism theory, which proposes the existence of different cortical pathways for slow- (<6^o^/s) and fast- (>22^o^/s) moving stimuli, highlighting the importance of area V5 in high-speed processing, and suggesting a supportive role for other cortical areas (64). However, since empirical findings are not always in line with this proposed ‘double dissociation’ of speed-detection areas (4, 47), the validity of the dynamic parallelism theory is likewise in need of further empirical testing.

### 4.2 Recommendations for clinical practice

The primary treatment options for akinetopsia are currently limited to practice-based methods aimed at alleviating either the underlying condition (mostly through medication) or its secondary effects. For example, some studies indicate potential for rehabilitation through restorative training of surviving components of visual motion perception (9, 32, 41). Compensatory strategies, such as learning to judge vehicle speed by sound, have also shown promise in improving daily functioning and promoting independence (4, 47). Obviously the potential successes of such interventions stand and fall with insight and motivation. If a patient is unaware of the existence or the extent of their deficit, or is not ready to accept it, they may become unmotivated or intolerant of diagnostic processes and interventions (9). Our review identified a few incidences of people unaware of their deficit until it resulted in negative consequences (i.e., phobia, car crash), highlighting that unawareness of this perceptual deficit is not uncommon and not without its dangers. Finally, insights into akinetopsia and acknowledgement of its consequences can help raise awareness of the condition across scientific, clinical, and societal domains, and may help to inform policymakers and healthcare providers about the need for support systems tailored to address the multifaceted challenges it poses. By prioritising the improvement of the quality of life of people with akinetopsia, we can strive towards a more inclusive and supportive environment for those effected. That said, presently the best thing to do in clinical practice is a careful workup, followed by treatment of any underlying conditions, restorative training of surviving capacities to perceive movement, and psychoeducation.

### 4.3 Recommendations for scientific research

The incidence and prevalence rates of akinetopsia are as yet unknown, but given the obscurity of this condition there may be many more undiagnosed individuals in society at large, unaware of what they are suffering from. Therefore, we would firstly recommend to screen clinical as well as nonclinical populations for akinetopsia, preferably with the aid of a standardised diagnostic tool. The condition’s unfamiliarity across societal, clinical, and scientific domains perpetuates the lack of standardised assessments and systematic investigations, especially when contrasted with the more extensive research dedicated to other perceptual deficits such as prosopagnosia (face blindness). This underscores the need for greater awareness, improved diagnostic criteria and assessment methods, and further scientific research into the neural circuitry of motion perception to better identify and understand the prevalence, variability and causes of akinetopsia. On the basis of the findings here presented we also recommend research into the mediating role of object velocity in akinetopsia, which might benefit from studying a wider range of velocities and a systematic assessment of their interactions with visual motion perception. Ideally, this should be done in double-dissociation studies, which have the potential to enhance our understanding of the motion-perception pathways and test the dynamic parallelism theory. More generally, we suggest that future studies on akinetopsia enhance the detail of phenomenological descriptions, and prioritise cross-sectional as well as longitudinal study designs while integrating protocols to ensure standardised documentation in larger and more diverse samples. Longitudinal clinical studies have the potential to provide valuable insights into the progression and prognosis of (hemi)akinetopsia, shedding light on symptom frequency, duration, and other aspects over time. Experimental studies, on the other hand, might compare the effects of unilateral and bilateral stimulation across different regions of the visual motion-perception network and incorporate neuroimaging techniques to map the neural correlates of the motion-perception network. For example, diffusion tensor imaging (DTI) or fMRI studies in healthy participants performing motion-perception tasks might inform us about the velocity at which area V5 becomes active, and further elucidate the roles of different components within the motion perception network as a whole. Developing an understanding of the impact of hemispheric lateralisation on the expression of akinetopsia could be used for rehabilitation strategies and potentially improve outcomes for affected individuals. This understanding could be enhanced through data analysis techniques such as lesion network mapping (LNM), which can identify critical brain regions and networks linked to the condition. This approach not only enables targeted therapeutic interventions but also aids to deepen insight into the factors that may mediate symptom heterogeneity (65). Inspired by the experimental studies cited, we also propose the development of a diagnostic psychophysical tool for akinetopsia. For example, a computerised display of various movement types combined with a verbal motion discrimination task could be used to identify visual motion-perception impairments. This tool would preferably offer an accessible as well as cost- and time-efficient alternative to existing methods. Finally, we recommend to probe the potential connection between akinetopsia on the one hand and motion-perception deficits in multiple sclerosis on the other. Studies suggest that motion-perception deficits in multiple sclerosis may arise from disruptions in visual processing pathways (66), with optic neuritis being the most commonly affected afferent visual pathway manifestation (67). This may result in difficulties in tracking moving objects or perceiving motion at certain speeds or in certain visual contexts. It is as yet unknown whether this may also present as akinetopsia, so it would be interesting whether there is a connection or perhaps an extension of the phenomenological continuum.

### 4.4 Limitations

Given the limited literature on akinetopsia, the present study had to rely on a modest number of publications which were moreover heterogeneous in design and which had sometimes used different names to address similar phenomena. This lack of uniformity and comprehensiveness across studies moreover precluded proper insight into such aspects as handedness, symptom frequency and duration, involvement of the visual hemifields, burden, and course. Our rather broad inclusion approach allowed us to uncover a rich array of clinical presentations of akinetopsia, but also made it harder to establish one-on-one correlations with brain lesions. Moreover, the TMS studies here reviewed lacked sufficiently detailed methodologies and well-optimised motion-detection tasks, limiting their capacity to demonstrate robust effects based on effect size and distribution.

## 5 Conclusion

Even if Stevens’s (1) adage that ‘life is motion’ is debatable, the present systematic review shows that motion *perception* is vital to everyday life. Drawing from our overview of 25 clinical and 27 experimental cases of akinetopsia, we conclude that this visual motion-perception impairment has far-reaching consequences for those affected, irrespective of its presentation as either continuous or intermittent, as affecting the whole field of vision or only half of it, or as becoming better or worse for different ranges of object velocity. We also conclude that it is being mediated by a diversity of factors (ranging from stroke to epilepsy and intoxications) that affect the cortical area V5 or the broader motion-perception network, with the right-sided area V5 (contrary to previous findings) being the most important hub in that network, even though its left-sided homologue was found to be most vulnerable to acute disturbances under experimental conditions. Our study moreover indicates that the phenomenological presentation of akinetopsia is influenced by a multitude of components, including, importantly, hemispheric lateralisation and object velocity. Due to a lack of data in the original studies, the burden of akinetopsia could not be assessed systematically, but individual cases confirm the condition’s negative impact on individuals’ functioning and well-being. Treatment options are currently limited to practice-based attempts to treat the underlying disorder, although studies in other areas (e.g., the agnosia) suggest that additional effects can be obtained with the aid of restorative training of surviving components of the visual motion-perception network. To overcome the gaps in understanding and to help people unaware of their condition to find their way to proper care, we need to raise awareness of akinetopsia and its burden in scientific, clinical, and societal spheres, thus stimulating further research, refining diagnostic approaches, developing novel therapeutic interventions, and improving the quality of life of those affected. Finally, even when nothing can be done in a practical sense, people with akinetopsia and those who care for them may be helped by knowing the name of their condition, by being reassured that it is not a mental disorder, by realising that one is not alone in experiencing this, and by mapping out in which ways this condition affects their daily functioning.

## Data Availability

The original contributions presented in the study are included in the article/Supplementary material, further inquiries can be directed to the corresponding author.

## References

[1] Stevens W. Harmonium: poems by Wallace Stevens. New York, NY: Alfred A. Knopf (1919).

[2] Blom JD. A dictionary of hallucinations. 2nd ed. Cham: Springer Nature (2023).

[3] Gilaie-Dotan S. Visual motion serves but is not under the purview of the dorsal pathway. Neuropsychologia. (2016) 89:378–92. doi: 10.1016/j.neuropsychologia.2016.07.018, PMID: 27444880

[4] Heutink J de Haan G Marsman J-B van Dijk M Cordes C. The effect of target speed on perception of visual motion direction in a patient with akinetopsia. Cortex. (2018) 119:511–8. 30661737 10.1016/j.cortex.2018.12.002

[5] Huberle E Rupek P Lappe M Karnath H-O. Perception of biological motion in visual agnosia. Front Behav Neurosci. (2012) 6:56. doi: 10.3389/fnbeh.2012.00056, PMID: 22973210 PMC3428581

[6] Haque S Vaphiades MS Lueck CJ. The visual agnosias and related disorders. J Neuroophthalmol. (2018) 38:379–92. doi: 10.1097/wno.0000000000000556, PMID: 28945627

[7] Zeki S. Cerebral akinetopsia (visual motion blindness). Brain. (1991) 114:811–24. doi: 10.1093/brain/114.2.811, PMID: 2043951

[8] Barton JJS. Disorders of higher visual processing. Neuroophthalmology. (2011) 102:223–61. doi: 10.1016/b978-0-444-52903-9.00015-7 21601069

[9] Burns MS. Clinical management of agnosia. Top Stroke Rehabil. (2004) 11:1–9. doi: 10.1310/n13k-ykyq-3xx1-nfav, PMID: 14872395

[10] Blom J. D. Alice in wonderland syndrome. Cham: Springer Nature (2020) 137–166.

[11] Cooper SA Joshi AC Seenan PJ Hadley DM Muir KW Leigh RJ . Akinetopsia: acute presentation and evidence for persisting defects in motion vision. J Neurol Neurosurg Psychiatry. (2011) 83:229–30. doi: 10.1136/jnnp.2010.223727, PMID: 21217160

[12] Zihl J von Cramon D Mai N. Selective disturbance of movement vision after bilateral brain damage. Brain. (1983) 106:313–40. doi: 10.1093/brain/106.2.313, PMID: 6850272

[13] Campbell R. Speechreading in the akinetopsic patient, L.M. Brain. (1997) 120:1793–803. doi: 10.1093/brain/120.10.1793, PMID: 9365371

[14] Pelak VS Hoyt WF. Symptoms of akinetopsia associated with traumatic brain injury and Alzheimer’s disease. Neuroophthalmology. (2005) 29:137–42. doi: 10.1080/01658100500218046

[15] Zihl J Heywood CA. The contribution of LM to the neuroscience of movement vision. Front Integr Neurosci. (2015) 9:6. doi: 10.3389/fnint.2015.00006, PMID: 25741251 PMC4330684

[16] Blanke O Landis T Mermoud C Spinelli L Safran AB. Direction-selective motion blindness after unilateral posterior brain damage. Eur J Neurosci. (2003) 18:709–22. doi: 10.1046/j.1460-9568.2003.02771.x, PMID: 12911768

[17] Schenk T Mai N Ditterich J Zihl J. Can a motion-blind patient reach for moving objects? Eur J Neurosci. (2000) 12:3351–60. doi: 10.1046/j.1460-9568.2000.00194.x, PMID: 10998118

[18] Van Swol JM Thompson EB Joffe JA Nguyen SA Berman EL. Akinetopsia: a systematic review. J Neuroophthalmol. (2024) 44:e483–8. doi: 10.1097/WNO.0000000000002032, PMID: 37938052

[19] Ardila A. Some unusual neuropsychological syndromes: somatoparaphrenia, akinetopsia, reduplicative paramnesia, autotopagnosia. Arch Clin Neuropsychol. (2016) 31:456–64. doi: 10.1093/arclin/acw021, PMID: 27193360

[20] Wagner W. Anisognosie, Zeitrafferphänomen und Uhrzeitagnosie als Symptome der Störungen im rechten Parieto-Occipitallappen. Nervenarzt. (1943) 16:49–56.

[21] Hoff H Pötzl O. Über die labyrinthären Beziehungen von Flugsensationen und Flugträumen. Eur Neurol. (1937) 97:193–211. doi: 10.1159/000148735

[22] Goldstein K Gelb A. Psychologische Analysen hirnpathologischer Fälle auf Grund von Untersuchungen Hirnverletzter: I. Abhandlung. Zur Psychologie des optischen Wahrnehmungs-und Erkenungsvorganges. Zeitschrift für die gesamte Neurologie und Psychiatrie. (1918) 41:1–142. doi: 10.1007/BF02874477, PMID: 39711969

[23] Blom JD Nanuashvili N Waters F. Time distortions: a systematic review of cases characteristic of alice in wonderland syndrome. Front Psych. (2021) 12:668633. doi: 10.3389/fpsyt.2021.668633, PMID: 34025485 PMC8138562

[24] Pötzl O Redlich E. Demonstration eines Falles von bilateraler Affektion beider Occipitallappen. Wien Klin Wochenschr. (1911) 24:517–8.

[25] Schreber DP. Denkwürdigkeiten eines Nervenkranken. Leipzig: Mutze (1903).

[26] Nawrot M. Disorders of motion and depth. Neurol Clin. (2003) 21:609–29. doi: 10.1016/s0733-8619(02)00126-3, PMID: 13677815

[27] Blom JD. Alice in wonderland syndrome: a systematic review. Neurol Clin Pract. (2016) 6:259–70. doi: 10.1212/CPJ.0000000000000251, PMID: 27347442 PMC4909520

[28] New JJ Scholl BJ. “Perceptual scotomas”: a functional account of motion-induced blindness. Psychol Sci. (2008) 19:653–9. doi: 10.1111/j.1467-9280.2008.02139.x 18727780

[29] Zeki S. Area V5 – a microcosm of the visual brain. Front Integr Neurosci. (2015) 9:21. doi: 10.3389/fnint.2015.00021, PMID: 25883556 PMC4381624

[30] Dukelow SP DeSouza JF Culham JC van den Berg AV Menon RS Vilis T. Distinguishing subregions of the human MT+ complex using visual fields and pursuit eye movements. J Neurophysiol. (2001) 86:1991–2000. doi: 10.1152/jn.2001.86.4.1991, PMID: 11600656

[31] Morrone MC Tosetti M Montanaro D Fiorentini A Cioni G Burr DC. A cortical area that responds specifically to optic flow, revealed by fMRI. Nat Neurosci. (2000) 3:1322–8. doi: 10.1038/81860, PMID: 11100154

[32] Barton JJS. Cerebral visual loss. Ann Indian Acad Neurol. (2022) 25:S106–12. doi: 10.4103/aian.aian_136_22, PMID: 36589033 PMC9795709

[33] Devinsky O Farah MJ Barr WB. Chapter 21 visual agnosia. Handb Clinical Neurol. (2008) 88:417–27. doi: 10.1016/s0072-9752(07)88021-3, PMID: 18631704

[34] ffytche DH Lappin JM Philpot M. Visual command hallucinations in a patient with pure alexia. J Neurol Neurosurg Psychiatry. (2004) 75:80–6. 14707313 PMC1757477

[35] Kraft A Grimsen C Kehrer S Bahnemann M Spang K Prass M . Neurological and neuropsychological characteristics of occipital, occipito-temporal and occipito-parietal infarction. Cortex. (2014) 56:38–50. doi: 10.1016/j.cortex.2012.10.004, PMID: 23206528

[36] Ovsiew F. The Zeitraffer phenomenon, akinetopsia, and the visual perception of speed of motion: a case report. Neurocase. (2014) 20:269–72. doi: 10.1080/13554794.2013.770877, PMID: 23557277

[37] Álvarez R Masjuan J. Agnosias visuales. Rev Clin Esp. (2016) 216:85–91. doi: 10.1016/j.rce.2015.07.009, PMID: 26358494

[38] Otsuka-Hirota N Yamamoto H Miyashita K Nagatsuka K. Invisibility of moving objects: a core symptom of motion blindness. BMJ Case Rep. (2014) 2014:2013201233. doi: 10.1136/bcr-2013-201233, PMID: 24729106 PMC3987254

[39] Sakurai K Kurita T Takeda Y Shiraishi H Kusumi I. Akinetopsia as epileptic seizure. Epilepsy Behav Case Rep. (2013) 1:74–6. doi: 10.1016/j.ebcr.2013.04.002, PMID: 25667833 PMC4150625

[40] Tsai P-H Mendez MF. Akinetopsia in the posterior cortical variant of Alzheimer disease. Neurology. (2009) 73:731–2. doi: 10.1212/wnl.0b013e3181b59c07, PMID: 19720982

[41] Becker HGT Haarmeier T Tatagiba M Gharabaghi A. Electrical stimulation of the human homolog of the medial superior temporal area induces visual motion blindness. J Neurosci. (2013) 33:18288–97. doi: 10.1523/jneurosci.0556-13.2013, PMID: 24227738 PMC6619749

[42] Martinaud O. Visual agnosia and focal brain injury. Rev Neurol. (2017) 173:451–60. doi: 10.1016/j.neurol.2017.07.009, PMID: 28843416

[43] Vaina LM Cowey A Eskew RT LeMay M Kemper TL. Regional cerebral correlates of global motion perception. Brain. (2001) 124:310–21. doi: 10.1093/brain/124.2.310, PMID: 11157558

[44] Boes AD Prasad S Liu H Liu Q Pascual-Leone A Caviness VS Jr . Network localization of neurological symptoms from focal brain lesions. Brain. (2015) 138:3061–75. doi: 10.1093/brain/awv228, PMID: 26264514 PMC4671478

[45] Holmström L Vollmer B Tedroff K Islam M Persson JK Kits A . Hand function in relation to brain lesions and corticomotor-projection pattern in children with unilateral cerebral palsy. Dev Med Child Neurol. (2010) 52:145–52. doi: 10.1111/j.1469-8749.2009.03496.x, PMID: 19807768

[46] Cooper SA O’Sullivan M. Here, there and everywhere: higher visual function and the dorsal visual stream. Pract Neurol. (2016) 16:176–83. doi: 10.1136/practneurol-2015-001168, PMID: 26786007

[47] Heutink J Indorf DL Cordes C. The neuropsychological rehabilitation of visual agnosia and Balint’s syndrome. Neuropsychol Rehabil. (2018) 29:1489–508. doi: 10.1080/09602011.2017.1422272, PMID: 29366371

[48] Maeda K. Akinetopsia on driving. J Stroke Cerebrovasc Dis. (2019) 28:e102–3. doi: 10.1016/j.jstrokecerebrovasdis.2019.02.036, PMID: 31036340

[49] Nawrot M Rizzo M. Chronic motion perception deficits from midline cerebellar lesions in human. Vis Res. (1998) 38:2219–24. doi: 10.1016/s0042-6989(97)00297-6, PMID: 9797981

[50] Shipp S de Jong BM Zihl J Frackowiak RSJ Zeki S. The brain activity related to residual motion vision in a patient with bilateral lesions of V5. Brain. (1994) 117:1023–38. doi: 10.1093/brain/117.5.1023 7953586

[51] Zihl J von Cramon D Mai N Schmid CH. Disturbance of movement vision after bilateral posterior brain damage: further evidence and follow up observations. Brain. (1991) 114:2235–52. doi: 10.1093/brain/114.5.2235, PMID: 1933243

[52] Marcar VL Zihl J Cowey A. Comparing the visual deficits of a motion blind patient with the visual deficits of monkeys with area MT removed. Neuropsychologia. (1997) 35:1459–65. doi: 10.1016/s0028-3932(97)00057-2, PMID: 9352523

[53] The Mathworks, Inc. MATLAB for Windows, Version 23.2.0.2515942 (R2023b). (2023). Available at: https://www.mathworks.com

[54] Blanke O Landis T Safran AB Seeck M. Direction-specific motion blindness induced by focal stimulation of human extrastriate cortex. Eur J Neurosci. (2002) 15:2043–8. doi: 10.1046/j.1460-9568.2002.02038.x, PMID: 12099910

[55] Beckers G Hömberg V. Cerebral visual motion blindness: transitory akinetopsia induced by transcranial magnetic stimulation of human area V5. Proc Biol Sci. (1992) 249:173–8. doi: 10.1098/rspb.1992.0100, PMID: 1360678

[56] Beckers G Zeki S. The consequences of inactivating areas V1 and V5 on visual motion perception. Brain. (1995) 118:49–60. doi: 10.1093/brain/118.1.49, PMID: 7895014

[57] Schenk T Ellison A Rice N Milner AD. The role of V5/MT+ in the control of catching movements: an rTMS study. Neuropsychologia. (2005) 43:189–98. doi: 10.1016/j.neuropsychologia.2004.11.006, PMID: 15707904

[58] Walsh V Ellison A Battelli L Cowey A. Task-specific impairments and enhancements induced by magnetic stimulation of human visual area V5. Proc R Soc B. (1998) 265:537–43. doi: 10.1098/rspb.1998.0328, PMID: 9569672 PMC1688918

[59] Barton JJS. Motion perception and its disorders. Handbook Clin Neurol. (2021) 178:257–75. doi: 10.1016/B978-0-12-821377-3.00013-1, PMID: 33832680

[60] Horton JC Trobe JD. Akinetopsia from nefazodone toxicity. Am J Ophthalmol. (1999) 128:530–1. doi: 10.1016/s0002-9394(99)00177-4, PMID: 10577608

[61] Maeda K Sugihara Y Shiraishi T. Akinetopsia with achromatopsia due to focal epilepsy. Seizure. (2019) 67:27–9. doi: 10.1016/j.seizure.2019.03.004, PMID: 30856459

[62] Heywood CA Zihl J. Motion blindness In: Humphreys GW, editor. Case studies in the neuropsychology of vision. Hove: Psychology Press (1999). 17–40.

[63] Vaina LM. Selective impairment of visual motion interpretation following lesions of the right occipito-parietal area in humans. Biol Cybern. (1989) 61:347–59. doi: 10.1007/BF00200800, PMID: 2790066

[64] Ffytche DH Guy CN Zeki S. The parallel visual motion inputs into areas V1 and V5 of human cerebral cortex. Brain. (1995) 118:1375–94. doi: 10.1093/brain/118.6.1375 8595471

[65] Fox D. Mapping symptoms to brain networks with the human connectome. N Engl J Med. (2018) 379:2237–45. doi: 10.1056/NEJMra1706158 30575457

[66] Ayadi N Oertel FC Asseyer S Rust R Duchow A Kuchling J . Impaired motion perception is associated with functional and structural visual pathway damage in multiple sclerosis and neuromyelitis optica spectrum disorders. Mult Scler. (2021) 28:757–67. doi: 10.1177/13524585211032801, PMID: 34379018 PMC8978464

[67] Costello F. Vision disturbances in multiple sclerosis. Semin Neurol. (2016) 36:185–95. doi: 10.1055/s-0036-1579692, PMID: 27116725

[68] Vaina LM Lemay M Bienfang DC Choi AY Nakayama K. Intact “biological motion” and “structure from motion” perception in a patient with impaired motion mechanisms: a case study. Vis Neurosci. (1990) 5:353–69. doi: 10.1017/S0952523800000444 2265150

[69] Lane TL Liou TH Kung YC Tseng P Wu CW. Functional blindsight and its diagnosis. Front Neurol. (2024) 15:1207115. doi: 10.3389/fneur.2024.1207115, PMID: 38385044 PMC10879618

[70] Rizzo M Nawrot M Zihl J. Motion and shape perception in cerebral akinetopsia. Brain. (1995) 118:1105–27. doi: 10.1093/brain/118.5.1105, PMID: 7496774

[71] Viscardi LH Diniz Kleber F Custódio H Brandelli Costa A Brollo J. Akinetopsia (visual motion blindness) associated with brain metastases: a case report. Neurol Sci. (2024) 45:4621–3. doi: 10.1007/s10072-024-07565-x 38691276

[72] Hess RF Baker CL Jr. Zihl J. The “motion-blind” patient: Low level spatial and temporal filters. J Neurosci. (1989) 9:1628–40. doi: 10.1523/JNEUROSCI.09-05-01628.1989 2723744 PMC6569833

[73] Heutink J. de Haan G. Marsman J.-B. van Dijk M. Cordes C. The effect of target speed on perception of visual motion direction in a patient with akinetopsia. Cortex. (2019) 119:511–8. doi: 10.1016/j.cortex.2018.12.002 30661737

[74] Nawrot M Rizzo M Rockland KS Howard M. A transient deficit of motion perception in human. Vision Res. (2000) 40:3435–46. doi: 10.1016/s0042-6989(00)00177-2 11058740

