# Supplementary material for: Akinetopsia: a systematic review on visual motion blindness
Source: Front Neurol. 2025 Feb 10;15:1510807. doi: 10.3389/fneur.2024.1510807 (PMC11847689; doi:10.3389/fneur.2024.1510807)
Supplement: Supplementary file 1 [file Data_Sheet_1.pdf]

Supplementary material for Browne et al. (2025)

Fig. S1 Heatmap for hemispheric involvement vs. frequency of akinetopsia

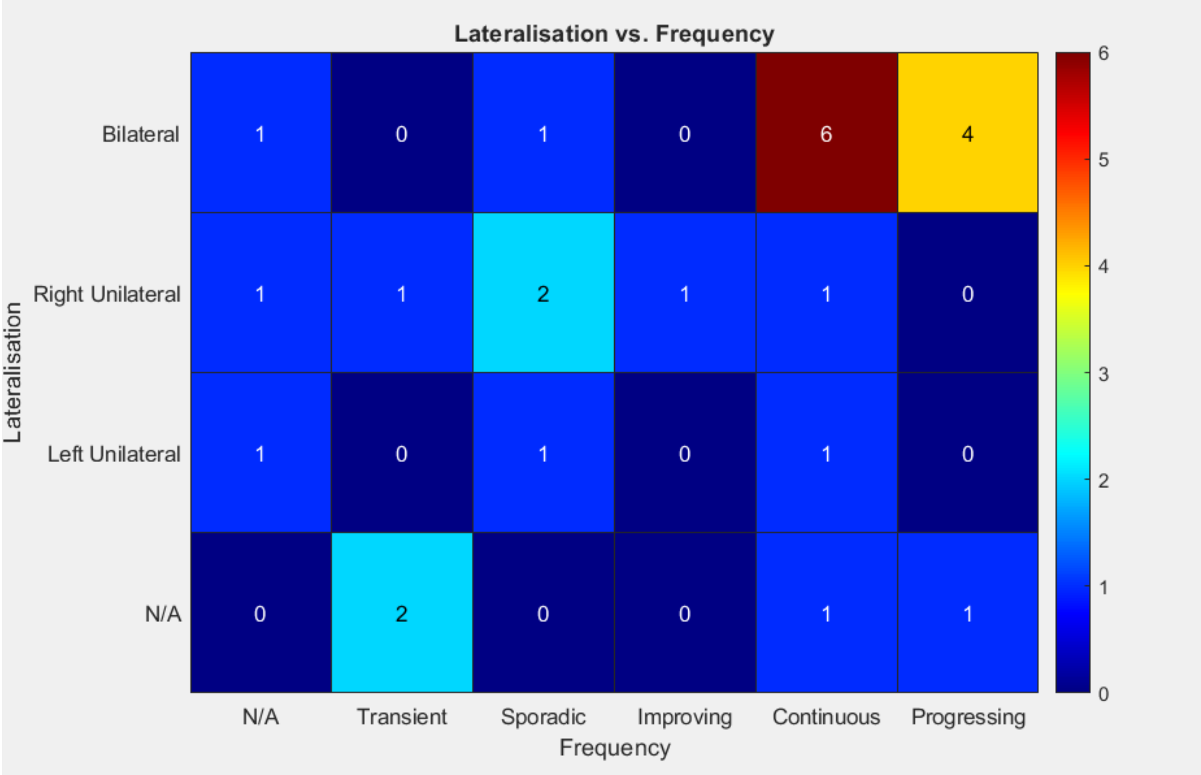

Fig. S2 Heatmap for hemispheric involvement vs. duration of akinetopsia

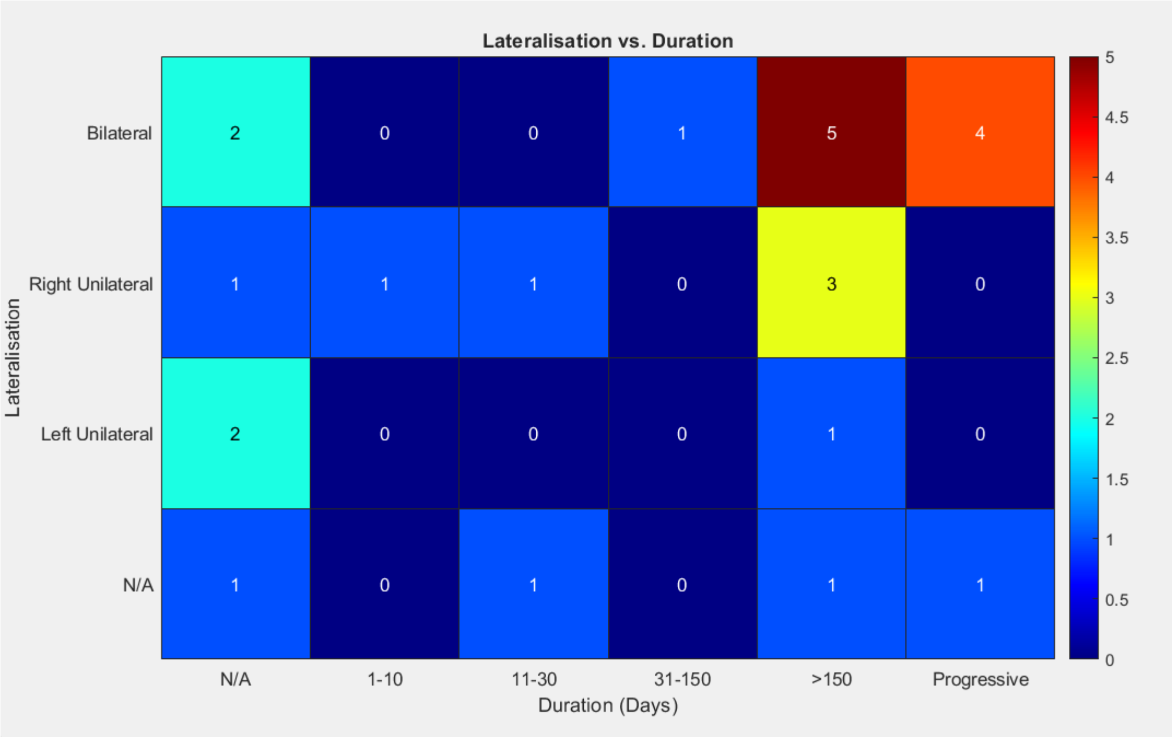

Fig. S3 Heatmap for hemispheric involvement vs. visual-field involvement in akinetopsia

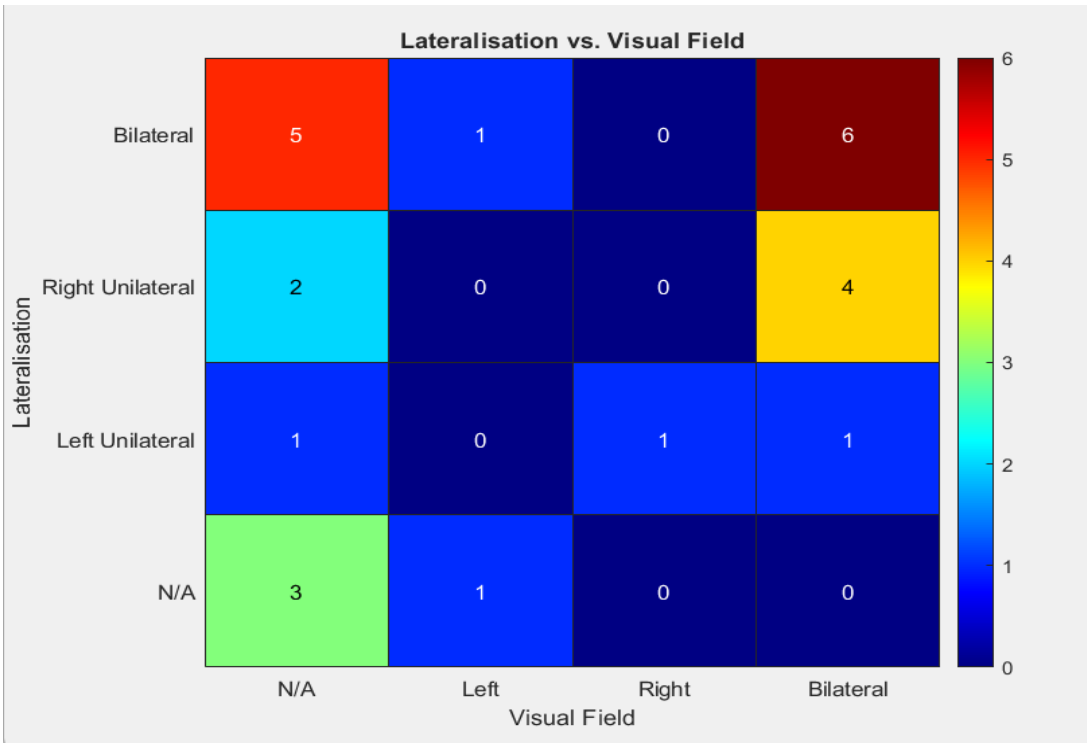

Table S1 Clinical cases of akinetopsia (n=25): Patient demographics and aetiological factors

Patient Name/ Label	Earliest Known Citation	Gender	Symptom Onset Age	Aetiological Factor	Affected Hemisphere	Impacted Lobule(s)	V5 Impairment
L.M	(Zihl et al., 1983)	Female	43	Stroke	Bilateral	Temporal & Occipital	Yes
A.F	(Vaina et al., 1990)	Male	60	Stroke	Bilateral	Temporal, Parietal & Occipital	N/A
Patient 1	(Cooper et al., 2011)	Female	61	Stroke	Right	Parietal & Occipital	No
Patient 2	(Cooper et al., 2011)	Male	56	Stroke	Left	N/A	Yes
T.D	(Heutink et al., 2018)	Female	37	Stroke	Bilateral	Occipital & Temporal	Yes
M.B	(Blanke et al., 2003)	Male	41	Stroke	Bilateral	Parietal	N/A
"54-year-old man"	(Maeda, 2019)	Male	54	Stroke	Right	Frontal, Temporal & Occipital	Yes
F.M	(Blanke et al., 2002)	Female	32	Seizure	Right	Temporal	N/A
"68-year-old woman"	(Maeda et al., 2019)	Female	68	Seizure	Bilateral	Parietal & Occipital	N/A
"A 61-year-old right-handed Japanese man"	(Sakurai et al., 2013)	Male	58	Seizure	Right	Temporal & Parietal	Yes
Case 1	(Tsai & Mendez, 2009)	Female	71	Neurodegeneration (PCA)	Bilateral	Parietal & Occipital	N/A
Case 2	(Tsai & Mendez, 2009)	Male	61	Neurodegeneration (PCA)	N/A	Parietal	N/A
Case 2	(Pelak & Hoyt, 2005)	Male	73	Neurodegeneration (PCA)	Bilateral	Temporal, Parietal & Occipital	N/A
"A 59-year-old woman"	(Cárdenas-Belaunzarán & Cerrillo-Avila, 2024)	Female	54	Neurodegeneration (PCA)	Bilateral	Parietal & Occipital	N/A
1 Nefazodone	(Horton & Throbe, 1999)	Male	47	Substances	N/A	N/A	N/A
2 Nefazodone	(Horton & Throbe, 1999)	Female	48	Substances	N/A	N/A	N/A
Schn	(Goldstein & Gelb, 1918)	Male	24	Trauma	Left	Parietal & Occipital	N/A
Case 1	(Pelak & Hoyt, 2005)	Male	58	Trauma	Bilateral	Occipital	N/A
JK	(Lane et al., 2024)	Male	20	Trauma	Bilateral	Occipital	N/A
"A right-handed Japanese man in his 60s"	(Otsuka-Hirota et al., 2014)	Male	60's	Surgery/ Subcortical Hemorrhage	Right	Temporal & Parietal	N/A
"A 68-year-old male"	(Naarden et al., 2019)	Male	68	Creutzfeld-Jakob Disease	Left	Parietal & Occipital	N/A
S.F	(Nawrot et al., 2000)	Female	19	Resection Surgery	Right	Temporal & Occipital	N/A
"a 49-year-old woman"	(Visquardi et al., 2024)	Female	49	Brain Metastases	Bilateral	Parietal, Occipital & Temporal	N/A
Mr. Janssen	(Blom, 2018)	Male	20's	N/A	N/A	N/A	N/A
B.L	(Pötzl & Redlich, 1911)	Female	58	N/A	Bilateral	Occipital	N/A

Table S2 Clinical cases of akinetopsia (n=25): phenomenology and interventions

Patient Name/ Label	Phenomenology	Symptom Frequency	Symptom Duration	Visual Field Presentation	Intervention
L.M	A loss of movement vision	Continious	>17 years	Bilateral	N/A
A.F	Impaired speed and idrection perception of movement	Continious	N/A	Left	N/A
Patient 1	Smooth movements were seen as a serioes of discontinious 'freeze frames'	Transient	5 days	Bilateral	N/A
Patient 2	Objects moving appeards to jump from one location to the next	Continious	>23 years	Right	N/A
T.D	Problems with perceiving movement	Continious	>8 months	N/A	N/A
M.B	Difficulties judging speed, direction and position of moving objects	Continious	A few months	N/A	N/A
"54-year-old man"	Impaired optic flow	N/A	N/A	N/A	N/A
F.M	Seizures were never accompanied by the perception of visual motion (and direction)	Sporadic	~ 10 years	N/A	N/A
"68-year-old woman"	During seizures what she was looking at would 'freeze' and loose colour, like a monochromic photo	Sporadic	> 1 year	Bilateral	200mg/day Carbamazepine
"A 61-year-old right-handed Japanese man"	Smooth movements suddenly resembles 'freeze frames'	Sporadic	>1 year	Bilateral	200mg/day Carbamazepine
Case 1	Akinetopsia when objects were moving left-wards	Progressing	Progressive	N/A	N/A
Case 2	Akinetopsia when objects were moving left-wards	Progressing	Progressive	Left	N/A
Case 2	Could no longer see motion 'while driving,: Motion Blindness	Progressing	Progressive	N/A	N/A
"A 59-year-old woman"	"Expiereinced difficulties with reading, … and walking by herself.."	Continious	Progressive	Bilateral	10 mg of donepezil daily, and began a cognitive rehabilitation programme
1 Nefazodone	Frozen images trailing in the wake of moving objects	Transient	N/A	N/A	Symptoms resolved after discontinuing nefazodone
2 Nefazodone	Motion parcellation	Transient	Few weeks (Feburary-March)	N/A	Reduced nefazodone dose at night from 400mg to 275mg.
Schn	Could not form definite impressions from movement (i.e., a hand moving from top to bottom would appear only on the top, and then the bottom, but the movements invetween would not be observed)	N/A	N/A	Bilateral	N/A
Case 1	Inability to perceive visual motion	Continious	>2 years	N/A	N/A
JK	" JK might be suffering from a form of akinetopsia, visual motion blindness, at least in the sense that for JK color and motion seem to dissociate, resulting in the appearance of a color patch that hovers motionless, as though viewing the individual frame of a film. "	Continious	>10 years	Bilateral (right)	Attempted rehabilitation
"A right-handed Japanese man in his 60s"	Moving objects were invisible to him following surgery	Continious	>5 months	Bilateral	N/A
"A 68-year-old male"	Sometimes failed to see any motion at all (akinetopsia)	Sporadic	N/A	N/A	N/A
S.F	Perception of motion was impaired	Gradual Improvement	A few weeks	Bilateral	N/A
"a 49-year-old woman"	"objects moving in front of her became static/frozen, as a cinematographic vision, she could only see their static image for a few seconds, even after they had been moved and later losing the sight of them"… "seeing the movement as a sequence of photos"	Continious	~11 days	Bilateral	N/A
Mr. Janssen	Object moving through his visual field seemingly ceased to exist temporarily, and were then recreated a fraction of a second later in a different location	Continious	>30 years	N/A	N/A
B.L	Visual perception was distrubed when it came to somewhat faster-moving objects	N/A	N/A	Bilateral	N/A

Table S3 Cases of experimentally-induced akinetopsia (n=27): overview

Study	(Becker et al., 2013)	(Blanke et al., 2002)	(Beckers & Hömberg, 1992)	(Beckers & Zeki, 1995)	(Shenk et al., 2005)	(Walsh et al., 1998)
**Stimulation**	Electrical Stimulation	Electrical Stimulation	TMS	TMS	rTMS	TMS
**N=**	1	1	8	5	6	6 (4 of which did all tasks)
**Age**						
Age	43	43				
Mean						
Range					21-38	6: (21-62), 4: (21-35)
Not specified			X	X		
**Gender**						
Male					3	
Female	1	1			3	
Not specified			X	X		X
**Handedness**						
Right		X			X (6)	X(6)
Left						
Not specified	X		X	X		
**Affliction**						
	left-temporal tumor	complex parietal seizures/ right temporal lobe epilepsy	N/A	N/A	N/A	N/A
**Cortical areas**						
MT/V5	X	X	X	X	X	X
MST	X					
V1			X	X		
Other	Angular gyrus			Control site: Non-V5 (~2cm posterior to V5)	Control Sites: Vertex & DS (Dorsal site; ~2cm dorsal to position of V5)	
**Lobule Stimulated**						
Occipital			V1 (Mid-occipital cortex, ~5cm above inion)	V1: (Occipital pole: various positions from 3-6cm above inion, up to 3cm laterally from the mid-sagital plane)		
Temporal		Lateral & Basal temporal lobe				
Parietal		X			DS: (Dorsal site; ~2cm dorsal to position of V5)	
Occipito-temporal	Temporo-occipito (anterior-posterior direction)					
Temporal-parietal						
Parieto-Occipito				Non-V5: prestriate cortex (2cm posterior to V5)	Vertex: intersection of mid-saggital plne and mid coronal plane	
Temporal-parietal-occipital			V5 (~5cm left/right of mid-saggital plane)	V5 (~6cm lateral from mid-saggital plne, &~3-4cm above mastoid-inion line)	V5: ~3cm above mastoid-inion line, & ~5cm lateral to mid-line in saggital plane)	V5: ~3cm above mastoid-inion line, & ~5cm lateral to mid-line in saggital plane)
**Hemispheric Stimulation**						
Bilateral			X (V1)	X (V1)	X (Vertex)	
Right Unilateral		X	X (V5 right)			
Left Unilateral	X		X (V5 left)	X (V5 & Non-V5)	X (V5 & DS)	X (V5)
**Visual Hemifield of Stimulus Presentation**						
Bilaterally					X	X
Left	X	X	X			
Right	X	X	X	X		
Central	X					
**Visual hemifield impairemnt**						
Both (Right & Left)	X				V5	
Left		X				
Right				V5		
Ipsilateral						
Contralateral			V5 (left/right)			
Effect present (not specified)				V1 (minimal effects)		
Not specified			V1 (Mid-occipital cortex, ~5cm above inion)	non- V5	V5 & Vertex	X
